# Interactive association of baseline and changes in serum uric acid on renal dysfunction among community‐dwelling persons

**DOI:** 10.1002/jcla.23166

**Published:** 2019-12-27

**Authors:** Ryuichi Kawamoto, Daisuke Ninomiya, Taichi Akase, Asuka Kikuchi, Teru Kumagi

**Affiliations:** ^1^ Department of Community Medicine Ehime University Graduate School of Medicine Toon-city Japan; ^2^ Department of Internal Medicine Seiyo Municipal Nomura Hospital Seiyo‐city Japan

**Keywords:** community‐dwelling person, eGFR, retrospective cohort study, serum uric acid

## Abstract

**Background:**

Chronic kidney disease (CKD) is a major public health concern. Baseline serum uric acid (SUA) levels were independently associated with incident renal dysfunction, but whether baseline and changes in SUA produce an interactive effect on renal dysfunction remains unclear.

**Methods:**

The subjects comprised 460 men aged 68 ± 10 (mean ± standard deviation) years and 635 women aged 68 ± 9 years from a rural village. We have found participants who underwent a similar examination 3 years later, and analyzed the relationship between baseline SUA, changes in SUA, and a 3‐year follow‐up renal function evaluated by estimated glomerular filtration rate (eGFR).

**Results:**

A total of 93 (20.2%) men and 76 (12.0%) women had hyperuricemia (men: SUA ≥ 7.0 mg/dL and women: SUA ≥ 6.0 mg/dL) at baseline. Multiple regression analysis using changes in eGFR as objective variable, adjusted for risk factors as explanatory variables, showed that the baseline SUA and changes in SUA were linearly associated with changes in eGFR (*β* = －0.115, *P* < .001 and *β* = －0.431, *P* < .001, respectively). In both normal SUA group and hyperuricemia group, changes in SUA significantly associated with changes in eGFR (*β* = －0.473, *P* < .001 and *β* = －0.197, *P* = .009, respectively). Participants with increased SUA from normal to hyperuricemia group had greater eGFR decline over the follow‐up period, and their multivariate‐adjusted 3‐year follow‐up eGFR was significantly lower than in other groups (*P* < .001).

**Conclusion:**

Our data demonstrated that baseline and longitudinal changes in SUA were independently and interactively associated with the renal function decline among community‐dwelling persons.

## 1 INTRODUCTION

The increasing prevalence of chronic kidney disease (CKD) is a common public health concern.^1^ In Japan, the prevalence of CKD increased significantly with time,^2^ and similar increases are being observed worldwide. There is now convincing evidence that persons with CKD and hyperuricemia have high rates of morbidity (eg, cardiovascular disease [CVD]), mortality,^3^, ^4^, ^5^ healthcare utilization,^6^, ^7^ and progression to end‐stage renal disease,^8^ for which systemic inflammation and oxidant stress are associated with a higher risk.^9^

For decades, high serum uric acid (SUA) levels were mainly considered a result rather than a cause of renal dysfunction.^10^ However, several experimental and epidemiological studies have demonstrated that increased SUA in humans is associated with systemic inflammation,^11^ hypertension,^12^ and progression to end‐stage renal disease.^13^ The main pathophysiological mechanisms of these deleterious effects caused by uric acid are endothelial dysfunction, activation of local renin‐angiotensin system, increased oxidative stress, and proinflammatory and proliferative actions.^14^ Moreover, changes in SUA from baseline to year 1 were also correlated with the decreased changes in the estimated glomerular filtration rate (eGFR).^15^ These studies provide direct evidence that SUA may be a true mediator of renal disease and progression. However, to our acknowledgment, few studies have examined whether baseline and changes in SUA have an interactive effect on the risk of renal dysfunction in the general Japanese population.

Thus, the aim of this study was to evaluate the relationship between baseline SUA, changes in SUA, and potential risk factors (ie, gender, age, body mass index [BMI], smoking habits, alcohol consumption, and prevalence of CVD, blood pressure, antihypertensive medication, triglycerides [TG], high‐density lipoprotein cholesterol [HDL‐C], low‐density lipoprotein cholesterol [LDL‐C], antilipidemic medication, hemoglobin A1c [HbA1c], and antidiabetic medication), and renal function by retrospective cohort data from community‐dwelling persons.

## 2 MATERIALS AND METHODS

### 2.1 Subjects

The present study was a retrospective cohort designed as part of the Nomura study.^16^ We have found and compared participants who underwent a similar examination 3 years later to this study. The study population was selected from those who received a community‐based annual health check at the Nomura Health and Welfare Center in rural areas in Ehime prefecture, Japan. Information on medical history, present conditions, and medications (antihypertensive [eg, thiazide diuretics], antilipidemic, antidiabetic, and uric acid–lowering [eg, allopurinol, feburic, and benzbromarone] medication) was obtained by interview using a structured questionnaire. Participants taking SUA‐lowering medication and with baseline eGFR < 15 mL/min/1.73 m^2^ were excluded. The study complies with the Declaration of Helsinki and was approved by the ethics committee of Ehime University School of Medicine with written informed consent obtained from each subject (Institutional Review Board: 1903018).

### 2.2 Evaluation of risk factors

Information on demographic characteristics and risk factors was collected using the clinical files of the subjects. BMI was calculated by dividing weight (in kilograms) by the square of height (in meters). Smoking status was defined as the number of cigarette packs per day multiplied by the number of years smoked (pack‐year), and the participants were classified into never smokers, past smokers, light smokers (<20 pack‐year), and heavy smokers (≥20 pack‐year). Daily alcohol consumption was measured using the Japanese liquor unit in which a unit corresponds to 22.9 g of ethanol, and the participants were classified into never drinkers, occasional drinkers (<1 unit/d), daily light drinkers (1‐2 units/d), and daily heavy drinkers (2‐3 units/d). We measured systolic blood pressure (SBP) and diastolic blood pressure (DBP) in the upper right arm of participants in the sedentary position using an automatic oscillometric blood pressure recorder, while the subjects were seated after having rested for at least 5 minutes. Appropriate cuff bladder size was determined at each visit based on arm circumference. TG, HDL‐C, LDL‐C, SUA, and HbA1c were measured during fasting. eGFR was calculated using CKD‐EPI equations modified by a Japanese coefficient: male, Cr ≤ 0.9 mg/dL, 141 × (Cr/0.9)^−0.411^ × 0.993 ^age^ × 0.813; Cr > 0.9 mg/dL, 141 × (Cr/0.9) ^−1.209^ × 0.993 ^age^ × 0.813; female, Cr ≤ 0.7 mg/dL, 144 × (Cr/0.7) ^−0.329^ × 0.993 ^age^ × 0.813; Cr > 0.7 mg/dL, 144 × (Cr/0.7) ^−1.209^ × 0.993 ^age^ × 0.813.^17^ Moreover, ischemic stroke, ischemic heart disease, and peripheral vascular disease were defined as CVD.

### 2.3 Statistical analysis

All values are expressed as the mean ± standard deviation (SD), unless otherwise specified, and in the cases of parameters with non‐normal distribution (such as TG and HbA1c), the data are shown as median (interquartile range) values. In all the analyses, parameters with non‐normal distributions were used after log‐transformation. Statistical analysis was performed using IBM SPSS Statistics Version 21 (Statistical Package for Social Science Japan, Inc). Hyperuricemia was defined as the level of SUA ≥ 7.0 mg/dL in men and ≥ 6.0 mg/dL in women.^18^ Three‐year changes in SUA and eGFR were calculated by subtracting the baseline values from those at 3 years. Participants were divided into four groups according to baseline and 3‐year follow‐up SUA (normal SUA throughout the follow‐up period, N → N group; hyperuricemia at baseline and normal SUA later, H → N group; normal SUA at baseline and hyperuricemia later, N → H group; and hyperuricemia throughout the follow‐up period, H → H group). Differences in means and prevalence among baseline and follow‐up findings were analyzed by paired *t* test for continuous data and chi‐square test for categorical data, respectively. Pearson's correlations were calculated in order to characterize the associations between various characteristics and changes in eGFR. Multiple linear regression analysis was used to evaluate the contribution of each confounding factor to changes in eGFR. ANCOVA was performed using a general linear model approach to determine the association between confounding factors and eGFR. In these analyses, eGFR was the dependent variable, the four categories according to baseline and a 3‐year follow‐up SUA were the fixed factors, and each confounding factor was added as covariates. The synergistic effect of baseline SUA category and changes in SUA on changes in eGFR was evaluated using a general linear model. A value of *P* < .05 was considered significant.

## 3 RESULTS

### 3.1 Baseline characteristics of participants categorized by baseline SUA

Baseline characteristics of participants categorized by baseline SUA are illustrated in Table 1. The subjects comprised 460 men aged 68 ± 10 (mean ± standard deviation) years and 635 women aged 68 ± 9 years from a rural village. The subjects were divided into two groups: normal SUA group and hyperuricemia group categorized by baseline SUA. The prevalence of men, BMI, smoking status, drinking status, the prevalence of CVD, DBP, the presence of antihypertensive medication, TG, the presence of antidiabetic medication, and SUA were significantly higher in the hyperuricemia group than in the normal SUA group, and HDL‐C, the presence of antilipidemic medication, and eGFR were significantly lower in the hyperuricemia group. There was no inter‐group difference regarding age, SBP, LDL‐C, and HbA1c.

**Table 1:** Baseline characteristics of participants categorized by baseline SUA

Baseline characteristics N = 1095	Normal SUA N = 926 Men: <7.0 mg/dL, women: <6.0 mg/dL	Hyperuricemia N = 169 Men: ≥7.0 mg/dL, women: ≥6.0 mg/dL	*P*‐value*
Men, N (%)	367 (39.6)	93 (55.0)	**<0.001**
Age (years)	68 ± 9	69 ± 9	0.386
Body mass index (kg/m^2^)	22.6 ± 3.0	23.4 ± 2.8	**0.001**
Smoking status^a^ (%)	74.5/16.7/2.8/5.9	62.7/26.6/3.6/7.1	**0.012**
Drinking status^b^ (%)	53.7/22.8/8.7/14.8	20.9/26.0/14.2/30.8	**<0.001**
Cardiovascular disease, N (%)	43 (4.6)	18 (10.7)	**0.005**
Systolic blood pressure (mmHg)	134 ± 17	136 ± 15	0.087
Diastolic blood pressure (mmHg)	77 ± 10	80 ± 10	**<0.001**
Antihypertensive medication, N (%)	374 (40.4)	88 (52.1)	**0.005**
Triglycerides (mg/dL)	85 (65‐117)	94 (69‐146)	**<0.001**
HDL cholesterol (mg/dL)	67 ± 17	63 ± 16	**0.011**
LDL cholesterol (mg/dL)	121 ± 29	116 ± 31	0.844
Antilipidemic medication, N (%)	219 (23.7)	38 (22.5)	**<0.001**
Hemoglobin A1c (%)	5.7 (5.4‐5.9)	5.7 (5.4‐6.0)	0.071
Antidiabetic medication, N (%)	71 (7.7)	17 (10.1)	**0.284**
eGFR (ml/min/1.73 m^2^)	74.3 ± 9.6	66.3 ± 15.0	**<0.001**
SUA (mg/dL)	4.9 ± 1.0	7.2 ± 0.8	**<0.001**

Abbreviations: eGFR, estimated glomerular filtration rate; HDL, high‐density lipoprotein; LDL, low‐density lipoprotein; SUA, serum uric acid.

a Smoking status was defined as the number of cigarette packs per day multiplied by the number of years smoked (pack‐year), and the participants were classified into never smokers, past smokers, light smokers (<20 pack‐year), and heavy smokers (≥20 pack‐year).

b Alcohol consumption was measured using the Japanese liquor unit in which a unit corresponds to 22.9 g of ethanol, and the participants were classified into never drinkers, occasional drinkers (<1 unit/d), daily light drinkers (1‐2 unit/d), and daily heavy drinkers (2‐3 unit/d). Data presented are mean ± standard deviation. Data for triglycerides and hemoglobin A1c were skewed and presented as median (interquartile range) values, and were log‐transformed for analysis.

* *P*‐value: Student's *t* test for continuous variables or the chi‐square test for categorical variables. Bolded numbers indicate significance.

### 3.2 Relationship between changes in SUA and changes in eGFR during the same period categorized by baseline SUA

**Figure 1:**
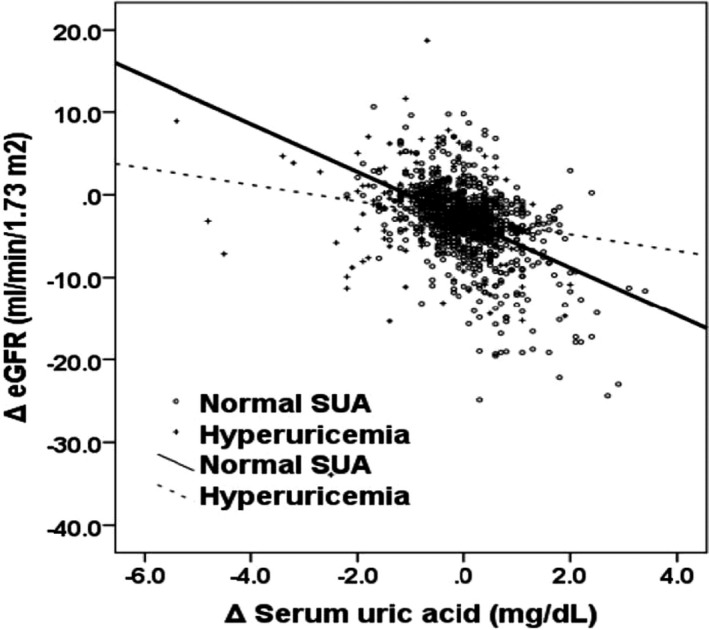
Relationship between changes in SUA and changes in eGFR during the same period categorized baseline SUA. In both normal SUA group (*r* = －.472, *P* < .001) and hyperuricemia group (*r* = －.184, *P* = .017), changes in SUA significantly correlated with changes in eGFR. But the regression line was stronger in the normal SUA group than in the hyperuricemia group, and analysis of covariance showed that two regression lines in each graph were significantly different (*F* = 27.88, *P* < .001)

As shown as Figure 1, in both normal SUA group (*r* = －.472, *P* < .001) and hyperuricemia group (*r* = －.184, *P* = .017), changes in SUA significantly correlated with changes in eGFR. But the regression line was significantly stronger in the normal SUA group than in the hyperuricemia group.

### 3.3 Relationship between baseline characteristics including changes in SUA and changes in eGFR categorized by baseline SUA

Table 2 shows the relationship between baseline characteristics including changes in SUA and changes in eGFR categorized by baseline SUA. In the normal SUA group, changes in SUA as well as age, HbA1c, and the presence of antidiabetic medication were negatively correlated with changes in eGFR, and in the hyperuricemia group, changes in SUA and eGFR were negatively correlated with changes in eGFR. While in total group, both SUA and changes in SUA as well as HbA1c, the presence of antidiabetic medication, and eGFR were correlated with changes in eGFR.

**Table 2:** Simple relationship between baseline characteristics including changes in SUA and changes in eGFR categorized by baseline serum uric acid

Baseline characteristics	Normal SUA N = 926 Men: <7.0 mg/dL, women: <6.0 mg/dL	Hyperuricemia N = 169 Men: ≥7.0 mg/dL, women: ≥6.0 mg/dL	Total N = 1095 Men: 0.6‐9.2 mg/dL, women: 0.9‐9.3 mg/dL
	*r* (*P*‐value*)	*r* (*P*‐value*)	*r* (*P*‐value*)
Gender (men = 0, women = 2)	.039 (.236)	.007 (.930)	.023 (.454)
Age	**−.068 (.040)**	−.016 (.835)	−.055 (.070)
Body mass index	−.036 (.278)	−.019 (.805)	−.023 (.440)
Smoking status	−.035 (.281)	−.008 (.919)	−.024 (.427)
Drinking status	−.027 (.407)	−.087 (.261)	−.022 (.465)
Cardiovascular disease (no = 0, yes = 1)	−.042 (.198)	.051 (.514)	−.010 (.743)
Systolic blood pressure	−.040 (.222)	−.122 (.115)	−.048 (.109)
Diastolic blood pressure	−.022 (.962)	.002 (.975)	.010 (.746)
Antihypertensive medication (no = 0, yes = 1)	−.060 (.070)	−.018 (.817)	−.044 (.150)
Triglycerides	−.020 (.535)	−.069 (.370)	−.020 (.504)
HDL cholesterol	.063 (.053)	.0542 (.504)	.054 (.075)
LDL cholesterol	.049 (.137)	.095 (.219)	.053 (.077)
Antilipidemic medication (no = 0, yes = 1)	**−**.022 (.510)	−.067 (.385)	−.031 (.306)
Hemoglobin A1c	**−.123 (<.001)**	.007 (.923)	**−.091 (.003)**
Antidiabetic medication (no = 0, yes = 1)	**−.068 (.039)**	−.056 (.471)	**−.062 (.040)**
eGFR	−.015 (.650)	**−.165 (.032)**	**−.076 (.012)**
SUA	.040 (.220)	.136 (.078)	**.096 (.002)**
Changes in SUA	**−.472 (<.001)**	**−.184 (.017)**	**−.400 (<.001)**

Note

Changes, 3‐y baseline; *r*, Pearson's correlation coefficient. Data for triglycerides and hemoglobin A1c were skewed and were log‐transformed for analysis.

* *P*‐value: Student's *t* test for continuous variables or the chi‐square test for categorical variables. Bolded numbers indicate significance.

### 3.4 Multivariate‐adjusted relationship between baseline characteristics including changes in SUA and changes in eGFR categorized by baseline SUA

Multiple regression analysis using changes in eGFR as an objective variable, adjusted for confounding factors as explanatory variables, showed that in the normal SUA group, changes in SUA as well as the presence of CVD, and HbA1c were significantly and independently associated with changes in eGFR (Table 3), and in the hyperuricemia group, changes in SUA as well age and eGFR were significantly associated with changes in eGFR. While in total group, both SUA and changes in SUA as well as age, HbA1c, and eGFR were significantly and negatively associated with changes in eGFR.

**Table 3:** Multivariate‐adjusted relationship between baseline characteristics including changes in SUA and changes in eGFR categorized by baseline SUA

Baseline characteristics	Normal SUA N = 926 Men: <7.0 mg/dL, women: <6.0 mg/dL		Hyperuricemia N = 169 Men: ≥7.0 mg/dL, women: ≥6.0 mg/dL		Total N = 1095 Men: 0.6‐9.2 mg/dL, women: 0.9‐9.3 mg/dL	
	Forced entry method	Stepwise method	Forced entry method	Stepwise method	Forced entry method	Stepwise method
	*β* (*P*‐value*)	*β* (*P*‐value*)	*β* *(P*‐value*)	*β* *(P*‐value*)	*β* *(P*‐value*)	*β* *(P*‐value*)
Gender (men = 0, women = 2)	−0.033 (.455)	**—**	0.136 (.297)	**—**	−0.020 (.622)	**—**
Age	**−0.147 (.001)**	**—**	−0.163 (.151)	**−0.225 (.020)**	**−0.158 (<.001)**	**−0.172 (<.001)**
Body mass index	−0.025 (.464)	**—**	0.000 (.996)	**—**	−0.008 (.799)	**—**
Smoking status	−0.033 (.353)	**—**	0.013 (.889)	**—**	−0.061 (.637)	**—**
Drinking status	−0.024 (.524)	**—**	−0.149 (.179)	**—**	−0.063 (.085)	**—**
Cardiovascular disease (no = 0, yes = 1)	**−0.061 (.036)**	**−0.067 (.019)**	0.022 (.785)	**—**	−0.030 (.282)	**—**
Systolic blood pressure	0.018 (.706)	**—**	**−0.261 (.032)**	**—**	−0.041 (.356)	**—**
Diastolic blood pressure	−0.005 (.913)	**—**	0.181 (.141)	**—**	0.027 (.532)	**—**
Antihypertensive medication (no = 0, yes = 1)	0.000 (.992)	**—**	−0.005 (.953)	**—**	0.009 (.778)	**—**
Triglycerides	0.021 (.536)	**—**	−0.044 (.623)	**—**	−0.003 (.921)	**—**
HDL cholesterol	0.030 (.384)	**—**	0.082 (.401)	**—**	0.039 (.239)	**—**
LDL cholesterol	−0.009 (.777)	**—**	0.025 (.775)	**—**	0.020 (.509)	**—**
Antilipidemic medication (no = 0, yes = 1)	−0.002 (.944)	**—**	−0.111 (.221)	**—**	−0.023 (.453)	**—**
Hemoglobin A1c	**−0.097 (.006)**	**−0.110 (<.001)**	0.162 (.100)	**—**	−0.052 (.121)	**−0.075 (.007)**
Antidiabetic medication (no = 0, yes = 1)	0.008 (.827)	**—**	−0.182 (.052)	**—**	−0.031 (.354)	**—**
eGFR	**−0.125 (.002)**	**—**	**−0.325 (.001)**	**−0.302 (.002)**	**−0.175 (<.001)**	**−0.180 (<.001)**
SUA	−0.072 (.052)	**—**	0.166 (.169)	**—**	**−0.083 (.029)**	**−0.115 (<.001)**
Changes in SUA	**−0.488 (<.001)**	**−0.473 (<.001)**	**−0.218 (.016)**	**−0.197 (.009)**	**−0.426 (<.001)**	**−0.431 (<.001)**
*R* ^2^	**0.255 (<.001)**	**0.240 (<.001)**	**0.195 (.012)**	**0.090 (.001)**	**0.199 (<.001)**	**0.191 (<.001)**

Note

Changes, 3‐y baseline; *β*, standard coefficient; *R* ^2^, multiple coefficient of determination. Data for triglycerides and hemoglobin A1c were skewed and were log‐transformed for analysis.

* *P*‐value: Bolded numbers indicate significance.

### 3.5 Comparison between baseline and the 3‐year follow‐up eGFR categorized by changes in SUA

**Figure 2:**
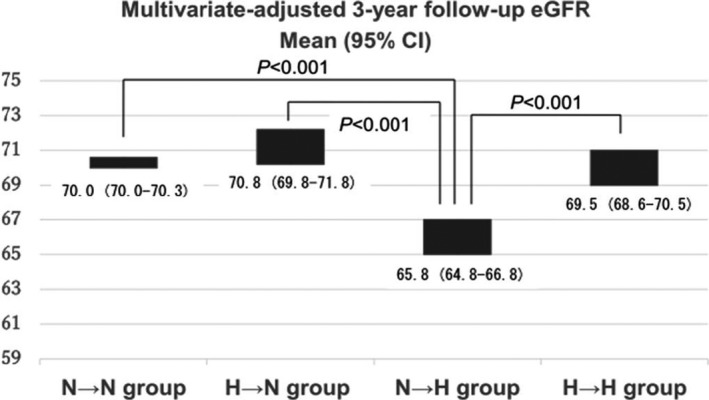
Multivariate‐adjusted 3‐y follow‐up eGFR categorized by changes in SUA. N‐N group participants with normal SUA throughout the follow‐up period, N‐H group those with normal SUA at baseline and hyperuricemia 3 y later, H‐N group those with hyperuricemia at baseline and normal SUA 3 y later, and H‐H group those with hyperuricemia throughout the follow‐up period

Furthermore, participants were divided into four groups for further analysis in Table 4. Eight hundred and fifty‐one participants had normal SUA throughout the follow‐up period (4.8 ± 1.0 mg/dL and 4.8 ± 1.0 mg/dL, respectively, defined as N‐N group); 77 participants had hyperuricemia at baseline and normal SUA later (7.1 ± 0.8 mg/dL and 5.8 ± 0.8 mg/dL, respectively, defined as H‐N group); 75 participants had normal SUA at baseline and had hyperuricemia later (5.8 ± 0.7 mg/dL and 7.0 ± 0.8 mg/dL, respectively, defined as N‐H group); and 92 participants had hyperuricemia throughout the follow‐up period (7.2 ± 0.9 mg/dL and 7.2 ± 0.7 mg/dL, respectively, defined as H‐H group). The eGFR significantly decreased in all groups. As shown in Figure 2, multivariate‐adjusted 3‐year follow‐up eGFR was significantly lower in the N → H group than in other groups, while there was no deference in between other groups.

**Table 4:** Comparison between baseline and 3‐y follow‐up eGFR categorized by changes in SUA

Characteristics N = 1095	N	eGFR			
		Baseline	3‐y follow‐up	Changes	*P*‐value*
N → N group	851	74.6 ± 9.3	71.7 ± 9.8	－2.9 ± 4.1	**<.001**
H → N group	77	66.8 ± 16.1	65.2 ± 16.7	－1.6 ± 6.1	**.024**
N → H group	75	71.2 ± 11.8	64.1 ± 14.9	－7.1 ± 6.1	**<.001**
H → H group	92	65.9 ± 14.2	63.3 ± 13.9	－2.6 ± 5.7	**<.001**

Note

N‐N group participants with normal SUA throughout the follow‐up period, N‐H group participants with normal SUA at baseline and hyperuricemia 3 y later, H‐N group participants with hyperuricemia at baseline and normal SUA 3 y later, and H‐H group participants with hyperuricemia throughout the follow‐up period.

* *P*‐value: Student's *t* test for continuous variables. Bolded numbers indicate significance.

## 4 DISCUSSION

In this retrospective cohort study of 1095 community‐dwelling persons, we set out to determine renal function, as assessed by eGFR, and examine potential confounding factors. Both baseline and changes in SUA were significantly and independently associated with changes in eGFR. This study demonstrated that the interactive relationship between baseline and changes in SUA was significantly associated with decreased changes in eGFR, adjusting for confounding factors including baseline eGFR and SUA. To our knowledge, few studies have indicated that baseline and changes in SUA could be important as a potential risk factor for renal dysfunction among community‐dwelling persons.

In subjects with normal renal function, an elevated SUA has almost uniformly been found to independently predict the development of CKD.^19^ Obermayr et al^20^ followed up 21 475 healthy volunteers for 7 years determined that after adjustment for multiple risk factors including baseline eGFR, age, sex, mean blood pressure, and metabolic syndrome, an elevated SUA level (7‐8.9 mg/dL) doubled the risk for incident kidney disease. Further, compared with those with lower SUA levels, those with SUA levels > 9 mg/dL had three times the risk for developing kidney disease. From a prospective cohort study following over 13 338 participants without renal dysfunction, Weiner et al^13^ found that a baseline elevated SUA level predicted worsening renal function irrespective of age, gender, race, diabetes, hypertension, alcohol use, smoking, lipids, and baseline renal function. Among over 6000 Japanese subjects, SUA levels of ≥5 mg/dL at initial screening of subjects with normal serum creatinine had a relative risk of 1.351 for developing high serum creatinine.^21^

Storhaug et al^22^ showed that an increase in SUA during follow‐up was associated with an increased risk of developing renal dysfunction after 7 and 13 years. Whelton et al^23^ in a study with febuxostat found that lowering SUA level 1 mg/dL from baseline improved eGFR by 1.15 mL/min/1.73 m^2^ (*P* < .001). In our study, each increased baseline and increased changes in SUA was significantly and independently associated with decreased changes in eGFR, and in addition, these effects were interactive. Thus, we think that it may be important to evaluate changes in SUA in addition to baseline SUA level when investigating the effect of SUA on renal dysfunction at follow‐up.^22^

To our knowledge, this study is the first to report an interactive effect between baseline and changes in SUA on the risk of renal dysfunction among community‐dwelling persons. The precise mechanisms are not completely understood. In addition to significant renal effects, excess accumulation of SUA can lead to various diseases.^23^ Recent epidemiological, experimental, and clinical studies have consistently showed that SUA has an important causative role in the onset and progression of hypertension,^8^, ^24^ diabetes,^25^ metabolic syndrome,^26^ CVD,^27^, ^28^ and CVD mortality.^29^ Moreover, a change in SUA level may be related to other lifestyle‐related factors, such as alcohol consumption, smoking status, socioeconomic stress, and exercise habits.^30^ Unfortunately, changes in such information are not evaluated in this study.

Uric acid is catalyzed by the enzyme xanthine oxidase, which is responsible for the production of uric acid and the damage of free radicals, and it also possesses dual pro‐oxidant and antioxidant properties. Uric acid is the final oxidation product of purine metabolism in humans and is renally excreted.^29^ Therefore, increased SUA levels are seen in participants with reduced GFR. However, in recent years, uric acid levels are inversely related to endothelial function,^31^ resulting in afferent arteriolar thickening and a decrease in vasodilatation^32^ which is known to be part of the pathophysiology of worsening renal function. Moreover, uric acid promotes apoptosis in human proximal tubule cells by oxidative stress and activation of NAPDH oxidase NOX 4 which might explain the chronic tubule‐interstitial damage observed in hyperuricemic states.^33^ In the analysis of longitudinal data, we found that participants with increased SUA from normal to hyperuricemia group had greater eGFR decline over the follow‐up period than in other groups. On the contrary, in participants with normal SUA at baseline, an increase in SUA level during follow‐up shows deterioration of renal function.

As a retrospective cohort study, this study had inevitable limitations. First, this study could not explain the causal relationship between hyperuricemia and renal function. Second, eGFR using the CKD‐EPI equation tends to be less accurate in subjects with normal renal function and CKD than GFR when inulin clearance is used, but is more accurate than serum creatinine or eGFR when the Modification of Diet in Renal Disease (MDRD) formula^17^ is used. Third, confounding factors and eGFR are based on a single assessment of blood, which may introduce a misclassification bias. Fourth, we could not eliminate the possible effects of underlying diseases and medications for hypertension, diabetes, and dyslipidemia on the present findings. Therefore, the demographics and referral source may limit generalizability.

In conclusion, this study showed that both baseline and increased changes in SUA contributed to eGFR decline, independent of baseline eGFR. The underlying mechanism behind this relationship is unknown, and these factors seem to be independent of confounding factors, such as age, gender, drinking status, smoking status, blood pressure, lipids, HbA1c, and medication. Further prospective population‐based studies are needed to investigate the changes in SUA metabolism and eGFR by lifestyle interventions.

## CONFLICT OF INTEREST

The authors declare that they have no competing interests.

## AUTHOR CONTRIBUTIONS

RK participated in the design of the study, performed the statistical analysis, and drafted the article. RK DN, TA, AK, and TK contributed to the acquisition and interpretation of data. RK, DN, and TK contributed to the conception and design of the statistical analysis. All authors read and approved the article.

## ETHICAL APPROVAL

All procedures performed in studies involving human participants were in accordance with the ethical standards of the institutional research committee at which the studies conducted (IRB Approval Number: 1903018).

## INFORMED CONSENT

We obtained consent through opt‐out procedure from all individual participants included in the study.

## Data Availability

The datasets analyzed in this study are available from the corresponding author (Ryuichi Kawamoto, rykawamo@m.ehime-u.ac.jp) on reasonable request.
